# Age relative to school class peers and emotional well-being in 10-year-olds

**DOI:** 10.1371/journal.pone.0214359

**Published:** 2019-03-26

**Authors:** Shuntaro Ando, Satoshi Usami, Tetsuya Matsubayashi, Michiko Ueda, Shinsuke Koike, Syudo Yamasaki, Shinya Fujikawa, Tsukasa Sasaki, Mariko Hiraiwa-Hasegawa, George Patton, Kiyoto Kasai, Atsushi Nishida

**Affiliations:** 1 Department of Psychiatry and Behavioral Science, Tokyo Metropolitan Institute of Medical Science, Tokyo, Japan; 2 Department of Neuropsychiatry, Graduate School of Medicine, The University of Tokyo, Tokyo, Japan; 3 Center for Research and Development on Transition from Secondary to Higher Education, The University of Tokyo, Tokyo, Japan; 4 Osaka School of International Public Policy, Osaka University, Osaka, Japan; 5 School of Political Science and Economics, Waseda University, Tokyo, Japan; 6 University of Tokyo Institute for Diversity and Adaptation of Human Mind (UTIDAHM), The University of Tokyo, Tokyo, Japan; 7 Department of Health Education, Graduate school of Education and Office for Mental Health Support, The University of Tokyo, Tokyo, Japan; 8 School of Advanced Sciences, SOKENDAI (The Graduate University for Advanced Studies), Kanagawa, Japan; 9 Center for Adolescent Health, Murdoch Children’s Research Institute, University of Melbourne, Victoria, Australia; International Telematic University Uninettuno, ITALY

## Abstract

**Purpose:**

The aim of this study was to investigate the effect of age relative to school (i.e., class or grade level) peers on emotional well-being and the role of possible mediators of this effect in early adolescence using a large set of individual-level data from a community survey.

**Methods:**

A large community-based survey of 10-year-old children and their primary parents was conducted in Tokyo, where the school entry cutoff date is fixed. Emotional well-being was assessed by the WHO (Five) Well-Being Index (WHO-5). Academic performance and the experience of being bullied at school were also evaluated as potential mediators of the effect of relative age.

**Results:**

A total of 4,478 children participated in the study. In a univariate linear regression analysis, the relative birthdate (continuous variable starting from the school entry date and ending at the last date of the academic grade) was negatively associated with emotional well-being (β = -0.043, *p* = .005). The path analyses suggested that academic performance and bullying mediated the relationship between the relative birthdate and emotional well-being (both *p* < .01).

**Conclusions:**

Among a sample of 10-year-olds, children who were younger compared to class peers had lower levels of emotional well-being. Academic performance and victimization by peers mediated the relationship.

## Introduction

Child and adolescent mental health is the issue of top priority globally. Mental health problems often have their onset in late childhood and adolescence [1] and are regarded as major causes of disease burden in young people globally [2]. In some, they are an antecedent to suicide [3], one of the leading causes of mortality among young people [4].

Age relative to peers may be an important environmental factor for mental health of adolescents because several studies have suggested an association between relative age and mental health in adolescents. These mental health indices include children’s feelings about school [5], peer problems [6], emotional symptoms [7], a referral to a psychology service [8], a psychiatric diagnosis [9], and suicide [10, 11]. However, the underlying mechanism of relative age effect on mental health has been unknown. To promote emotional wellbeing by reforming educational and social systems and policies, we need an evidence base for the underlying factors of relative age effect.

Several studies have shown disadvantages of being relatively younger than classmates in some aspects other than mental health. A relatively young age was also associated with poorer educational achievement [12, 13], sports performance [14], and bullying [5]. Since these are the disadvantages that may contribute to low confidence and self-esteem, they may be mediators of the effect of relative age on mental health. However, no study has examined whether the relative age effect on mental health is mediated by school-related disadvantages, including academic achievement and bullying.

The main objective of this study was to investigate whether age relative to class peers is related to emotional well-being in early adolescence. To address this issue, we used data from Japan, where the school entry cutoff date is fixed and no flexibility for school entry is allowed. Furthermore, we aimed to explore potentially mediating factors in the effect of relative age on emotional well-being. We hypothesized that poorer academic achievement and experiences of bullying could mediate the relationship between age relative to peers and emotional well-being [15, 16].

## Materials and methods

This study used data from a large-scale population-based survey (Tokyo Early Adolescence Survey [T-EAS]), which was treated as the first wave of a longitudinal cohort study (Tokyo Teen Cohort study [TTC]) [17]. The survey was community-based, and participants were recruited randomly from three municipalities in Tokyo using the resident register. The sample of this survey included 10-year-old children born between September 2002 and August 2004 and their primary parents. There is no cultural difference between the three neighboring municipalities, and all public schools in Japan operate under the same rules for the evaluation of academic achievement and bullying prevention. Therefore, there was no concern regarding potential clustering of data by municipality or school/classroom. Invitation letters were sent home corresponding to the birthdays of the intended participants, and trained interviewers visited their homes after the letters were sent. The interviewers explained the purpose and methodology of the study and obtained written informed consent from primary parents. Data were collected via a self-report questionnaire and face-to-face interviews for children and primary parents during home visits. For sensitive questions regarding bullying, a sealed envelope was provided for the self-report questionnaire to allow participants to answer confidentially.

### Dependent variable

#### Emotional well-being of children

The WHO-Five Well-Being Index (WHO-5) was applied for children to evaluate the emotional well-being of children. It contained five questions and used a 6-point Likert scale to explore mood and daily activities during the previous two weeks [18]. The validity of WHO-5 has already been shown for adolescents [19]. The items included the following: “I have felt cheerful and in good spirits,” “I have felt calm and relaxed,” “I have felt active and vigorous,” “I woke up feeling fresh and rested,” and “My daily life has been filled with things that interest me.” The raw score for each question (0 = not present to 5 = constantly present) was added up and transformed into a total score ranging from 0 (worst level of well-being) to 100 (best level of well-being).

### Independent variables

#### Relative birthdates of children

In Japan, the academic year starts at the beginning of April and ends at the end of March. Children are automatically assigned to an academic grade for elementary and junior high school according to their birthdays. Because April 2 is the starting birthdate and April 1 is the last birthdate used to determine assignment to an academic grade, birthdate was regarded as a continuous variable starting from April 2 and ending April 1 (ranging from 1 to 365).

#### Candidate mediators that explain the association between relative age and well-being

The candidate factors that underpin the association between relative birthdate and emotional well-being were chosen based on previous studies revealing certain factors influenced by the relative age of children. Because several previous studies showed that the academic achievements of relatively young children were less than those of older children [12, 13], academic performance at school was treated as a candidate for mediator of the relative age effect. Because physical strength was thought to be a risk for being bullied [15, 16] and relatively young children are physically small for their school grade, the experience of being bullied was also suggested as a candidate for mediator of the relative age effect.

#### Academic achievement at school

Primary parents evaluated children’s academic achievement at school using a 5-point Likert scale for each of five academic fields: mathematics, language, science, social studies, and physical education. In Japan, all public elementary schools issue a report card for each student evaluating achievement in each subject by the same standards and absolute scale. Report cards are issued twice a year, and parents must sign and return them to the school. The following is an example of questions asked of primary parents: “Please evaluate your child’s academic achievement in mathematics at school.” The response choices were *bad*, *relatively bad*, *normal*, *relatively good*, and *good*, and a score of 1 to 5 was given for each answer (a higher score meant better academic achievement). With regard to comparisons with other children, there is a parents’ day once a month, and parents are invited to visit classes and observe other children learning. Students receive their report cards in school at the same time, and they usually exchange and look at others’ report cards and tell their parents about them. Thus, we assumed that parents could evaluate their children’s academic achievements observationally to some extent. Because observational academic performance in each subject was associated significantly with both relative birthdate and emotional well-being, showing a high correlation with each other (Cronbach’s α = .85), scores for each subject were added up to form a total score regarding observational academic performance.

#### Experience of being bullied at school

Both children and their primary parents were asked about any experiences of children being bullied using the self-report questionnaire and sealed envelope. Due to setting-specific nature of bullying, collecting data on bullying involvement from multiple informants has been recommended [20]. Therefore, either children who reported an experience of being bullied at school or children whose primary parents reported the experience were defined as those who had an experience of being bullied at school. A part of the Olweus Bully/Victims Questionnaire was modified and used for assessing the experience of being bullied [21]. In the self-report questionnaire, the definition of bullying was given as follows: “A child is being bullied when another child, or a group of children, say or do mean and hurtful things to him or her. It is also bullying when a child is teased repeatedly in a way he or she doesn’t like. But it is not bullying when two children of about the same strength quarrel or fight.” For children, a dichotomous question used for assessment of the experience of being bullied was, “Have you ever been bullied at school?” Children who replied positively were defined as those who had experiences of being bullied. For primary parents, the question used for the assessment of the experience of being bullied was the following: “How often has your child been bullied at school in the past couple of months?” The response choices were *never*, *once or twice in two months*, *twice or three times a month*, *once a week*, and *several times a week*. Responses suggesting the frequency of more than *once or twice in two months* were regarded as experiences of being bullied.

#### Other variables

Potential confounders used in the multivariate analysis included age at time of survey, sex, height, child’s IQ, annual household income, and harsh parenting. The height of each child was measured at home by the interviewer. Child’s IQ was estimated using a short version of the Wechsler Intelligence Scale for Children (WISC-III), consisting of two subsets (Information and Picture Completion) [22]. Because the two-item version provided enough reliability and validity using pooled sample data in the 1990s [22], we developed an original formula for estimating IQ to fit T-EAS data with specific regional and period characteristics. For 28 children from the T-EAS participants, we conducted subsequent measurements of the full version of WISC-III by expert psychologists one year after the initial survey. Then, we revised a formula for estimating IQ, which had an acceptable reliability of measurement for Intelligence and Picture Completion (Cronbach α = .70 and .54, respectively); the calculated IQ from the formula including only the two subsets explained 78% of the IQ from the full version of WISC-III. Annual household income was reported by primary parents using the self-report questionnaire and sealed envelope. We assumed that parents could be harsh to children who exhibited low academic achievement. Therefore, harsh parenting was considered as a candidate for potential confounders, because a previous study on adolescent families suggested the association between harsh parenting and children’s depressive symptoms [23]. Two different types of harsh parenting were asked of primary parents, including adherence to parental corporal punishment and scolding children aloud. The following questions were used to assess parental corporal punishment and scolding aloud, respectively: “Do you slap your child as a means of discipline?” and “Do you scold your children aloud?” The response choices to each question were: *rarely*, *sometimes*, *often*, and *always*. These four responses were scored from 1 to 4, respectively. Because the two types of harsh parenting showed a moderate correlation (*r* = .514, *p* < .001), they were combined to form a total score for harsh parenting. We obtained ethical approval for this study from the research ethics committees of the research institutes that collaboratively conducted this study (Tokyo Metropolitan Institute of Medical Science, The University of Tokyo, and SOKENDAI).

### Statistical analysis

First, a linear regression analysis using subjective well-being as a dependent variable and children’s relative birthdate as an independent variable was applied. Second, a multiple linear regression analysis was conducted to control for the effect of potential confounders. Third, the candidate mediators for relative age effect (i.e., academic performance and experience of being bullied at school) were added to the multivariate regression analysis. To examine sex differences in the association, the regression model was fitted separately for each sex, and regression coefficients were compared between the two estimates using the z-statistic. Further, for the variables showing sex differences in regression coefficients, we added an interaction term into the final model and tested whether the interaction term was significant. In addition, a path analysis was used to examine the mediating effect of the candidate mediating factors between relative birthdate and emotional well-being. Model fit was assessed by a comparative fit index (CFI) and the root mean square error of approximation (RMSEA). To examine the sex difference in the path analysis, we conducted multiple group analysis and compared models with constrained and unconstrained estimates. Since the sample size was enough large for its appropriate use, the Sobel test was performed to test the presence of assumed mediation effects. All statistical analyses were conducted using SPSS version 22 and Amos 20.0 (IBM Corp, New York, USA).

## Results

A total of 4,478 children randomly chosen from the resident register in the three municipalities in Tokyo participated in the Tokyo Early Adolescence Survey (T-EAS) (response rate 43.8%). The demographic characteristics of the participants are shown in Table 1. As a result of hierarchical linear modeling, there was no evidence of difference in academic achievement and bullying experience between three municipalities (both ICC < .01). Correlations between all variables were examined, and there was no correlation between relative age and height (Table 2: *r* = -.025, *p* = .092).

**Table 1:** Demographic characteristics of the participants (n = 4,478).

	N/mean	%/SD
Relative birthdate	183.3	104.3
Female	2100	46.9
Height (cm)	137.7	6.2
IQ	107.6	14.2
Academic achievement at school^a^	18.9	3.7
Experience of being bullied at school	1629	36.7
Emotional well-being^b^	78.8	16.7
Annual household income (ten thousand yen)		
0–499	723	16.9
500–699	880	20.6
700–999	1301	30.5
1000-	1365	32
Harsh parenting^c^		
corporal punishment	1.5	0.6
scolding aloud	2.4	0.8

^a^ higher score means better academic achievement (ranging from 5 to 25)

^b^ higher score means better emotional well-being (ranging from 0 to 100)

^c^ higher score means more frequent harsh parenting (ranging from 1 to 4)

**Table 2:** Correlation table between all variables (upper figure represents Pearson’s *r* and lower Italic figure shows *p-value*).

	Relative age	Emotional well-being	Academic achievement	Experience of being bullied	Age	Sex	Height	IQ	Annual household income	Harsh parenting
Relative age	1	-.044**	-.161**	.044**	-.072**	.017	-.025	.044**	-.014	.039**
		.*003*	.*000*	.*003*	.*000*	.*253*	.*092*	.*003*	.*371*	.*009*
Emotional well-being	-.044**	1	.143**	-.173**	-.009	.031*	.012	.010	.024	-.046**
	.*003*		.*000*	.*000*	.*571*	.*036*	.*411*	.*516*	.*119*	.*002*
Academic achievement	-.161**	.143**	1	-.119**	-.011	.052**	.058**	.380**	.218**	-.161**
	.*000*	.*000*		.*000*	.*484*	.*001*	.*000*	.*000*	.*000*	.*000*
Experience of being bullied	.044**	-.173**	-.119**	1	-.025	-.138**	-.044**	.004	-.034*	.098**
	.*003*	.*000*	.*000*		.*096*	.*000*	.*004*	.*802*	.*027*	.*000*
Age	-.072**	-.009	-.011	-.025	1	-.022	.257**	-.043**	.023	-.025
	.*000*	.*571*	.*484*	.*096*		.*138*	.*000*	.*004*	.*129*	.*090*
Sex	.017	.031*	.052**	-.138**	-.022	1	.026	-.082**	.025	-.060**
	.*253*	.*036*	.*001*	.*000*	.*138*		.*087*	.*000*	.*103*	.*000*
Height	-.025	.012	.058**	-.044**	.257**	.026	1	.038*	.069**	-.031*
	.*092*	.*411*	.*000*	.*004*	.*000*	.*087*		.*011*	.*000*	.*037*
IQ	.044**	.010	.380**	.004	-.043**	-.082**	.038*	1	.216**	-.033*
	.*003*	.*516*	.*000*	.*802*	.*004*	.*000*	.*011*		.*000*	.*025*
Annual household income	-.014	.024	.218**	-.034*	.023	.025	.069**	.216**	1	-.057**
	.*371*	.*119*	.*000*	.*027*	.*129*	.*103*	.*000*	.*000*		.*000*
Harsh parenting	.039**	-.046**	-.161**	.098**	-.025	-.060**	-.031*	-.033*	-.057**	1
	.*009*	.*002*	.*000*	.*000*	.*090*	.*000*	.*037*	.*025*	.*000*	

* p-value < .05,

** p-value < .01

**Fig 1:**
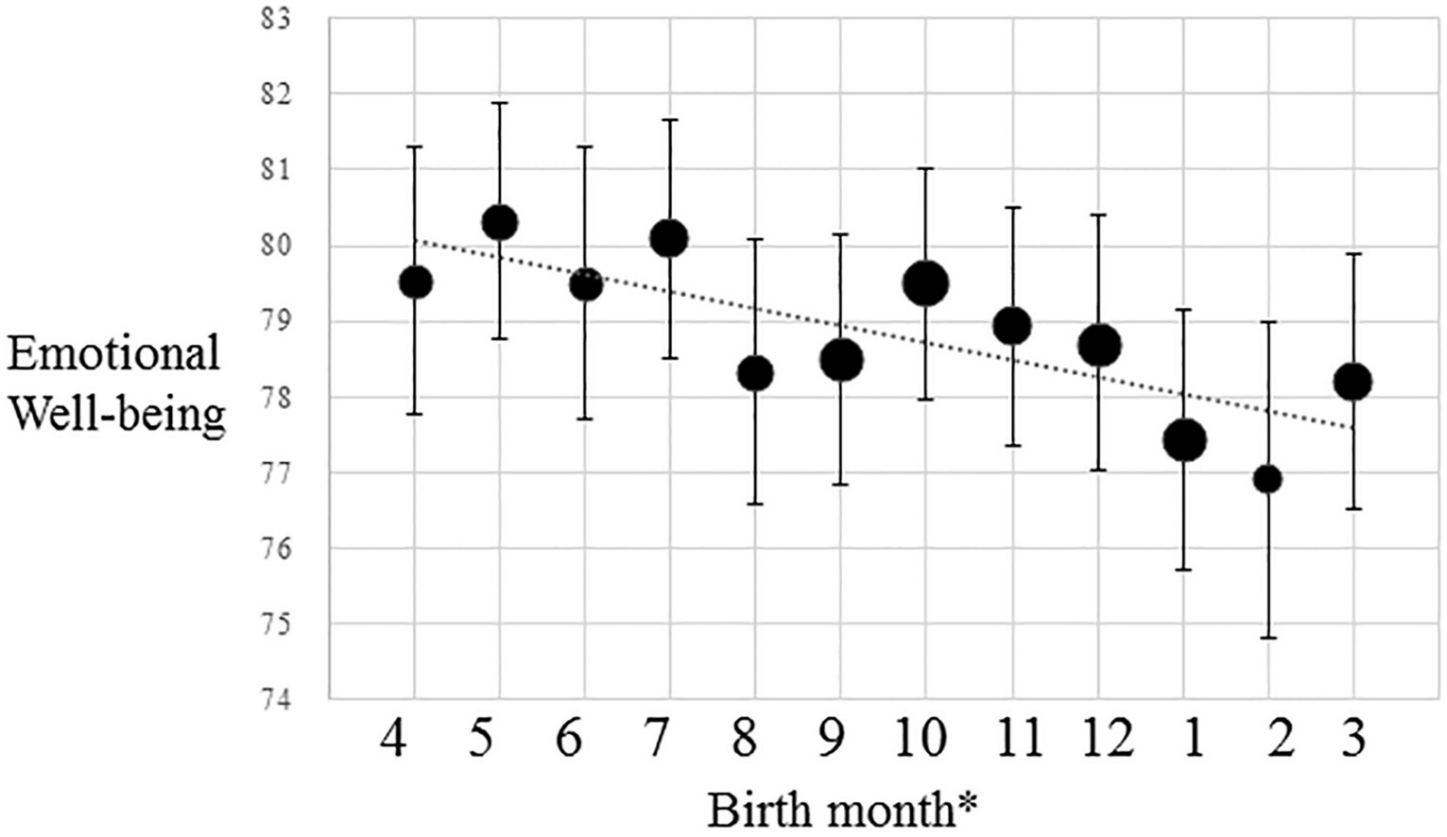
Birth month and emotional well-being. *Individuals born in 1^st^ April was treated as born in March.

**Fig 2:**
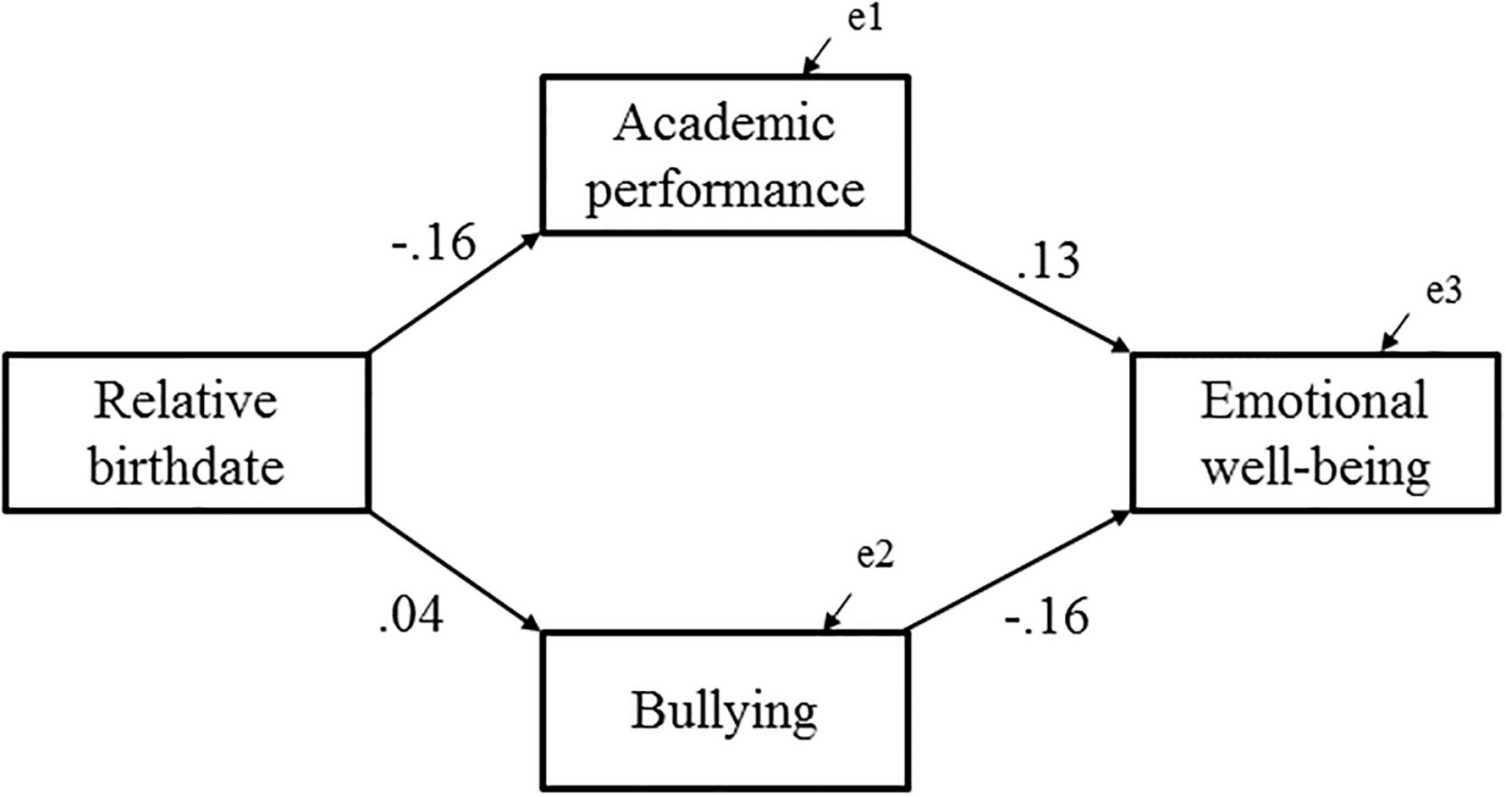
Path diagram describing the potential mediating effect of academic performance and bullying on the relationship between relative birthdate and emotional well-being. A residual correlation is assumed between academic performance and bullying. Note. Solid black line: path coefficient (*p* < 0.005). CFI = 0.999, RMSEA = 0.004, AIC = 27.264, BCC = 27.273.

A regression analysis showed the inverse association between relative birthdate and emotional well-being, which meant that relatively younger children in school grades had lower levels of emotional well-being (β = -0.043, *p* = .004; Table 3, Fig 1). This meant that about 104 days of relatively later birthdates would lead to a 0.7-point decrease in emotional well-being. The post-hoc statistical analysis showed that there was no interaction between sex and relative age on emotional well-being (*p* = .682). A multivariate linear regression analysis controlling for the effect of age at survey, sex, height, IQ, and annual household income still showed the significant effects of relative age on emotional well-being (β = -0.045, *p* = .003; Table 3). Further, we added academic performance at school and the experience of being bullied as candidate mediators of the relative age effect to the multivariate analysis. As a result of this analysis, academic performance at school (β = 0.137, *p* < .001) and the experience of being bullied (β = -0.156, *p* < .001) had significant associations with emotional well-being, whereas the significant association between relative birthdate and emotional well-being diminished (β = -0.013, *p* = .396). The post-hoc statistical analysis for interaction with sex indicated that the effect of bullying victimization on emotional well-being was larger in males than in females (*p* = .002; Table 4). Further, when the sexXbullying interaction term was added to model 4, the interaction term showed statistical significance (*p* < .05). The model assuming predictive pathways from academic achievement and bullying to well-being (Fig 2) showed good model fit (CFI = .999, RMSEA = .004). When examining sex difference by multiple group analysis, the model with constrained estimates on the path from bullying to well-being showed higher AIC and BCC (AIC = 61.519 and BCC = 61.631) than the model with unconstrained estimates (AIC = 53.615 and BCC = 53.732). In the analysis model (Fig 2), the Sobel test showed that the mediation effects of academic performance and bullying on the relationship between relative birthdate and emotional well-being were significant (standardized indirect effect = -.020 and -.007, respectively, both *p* < .01). The statistical analysis suggested that academic performance and being bullied had mediating effects on the association between relative birthdate and emotional well-being.

**Table 3:** The association between relative birthdate and emotional well-being considering candidate mediators.

	Unadjusted model		Model 1		Model 2		Model 3		Model 4	
	R^2^ = .002		R^2^ = .005		R^2^ = .024		R^2^ = .033		R^2^ = .047	
			ΔR^2^ = .004*		ΔR^2^ = .018**		ΔR^2^ = .027**^c^		ΔR^2^ = .014**	
	β^a^	p-value	β	p-value	β	p-value	β	p-value	β	p-value
Relative birthdate^b^	**-0.043**	**0.004**	**-0.045**	**0.003**	-0.017	0.294	**-0.039**	**0.010**	-0.013	0.396
Age			-0.014	0.387	-0.008	0.611	-0.016	0.311	-0.01	0.521
Sex			0.025	0.105	0.019	0.217	0.002	0.886	-0.001	0.955
Height			0.011	0.496	0.009	0.554	0.003	0.854	0.002	0.878
IQ			0.006	0.727	**-0.052**	**0.002**	0.006	0.691	**-0.044**	**0.010**
Annual household income			0.018	0.255	-0.002	0.899	0.014	0.375	-0.003	0.835
Harsh parenting			**-0.042**	**0.006**	-0.023	0.149	-0.025	0.102	-0.009	0.553
Academic performance at school					**0.155**	**<0.001**			**0.137**	**<0.001**
Experience of being bullied at school							**-0.169**	**<0.001**	**-0.156**	**<0.001**

^a^ Standardized regression coefficients

^b^ continuous variable starting from April 2nd (scored as 1) and ending at April 1st (scored as 365)

^c^ compared with Model 1

* p < .05,

** p < .01

**Table 4:** Sex difference in the association between relative birthdate and emotional well-being considering candidate mediators.

	Adjusted model for male		Adjusted model for female		Sex difference
	β^a^	p-value	β^a^	p-value	p-value
Relative birthdate^b^	-0.007	0.745	-0.019	0.403	0.650
Age	-0.016	0.455	-0.001	0.951	0.323
Height	0.031	0.144	-0.027	0.261	0.967
IQ	-0.054	0.020	-0.030	0.218	0.244
Annual household income	-0.028	0.189	0.025	0.284	0.046
Harsh parenting	-0.01	0.643	-0.01	0.675	0.493
Academic performance at school	0.158	<0.001	0.113	<0.001	0.916
Experience of being bullied at school	-0.198	<0.001	-0.101	<0.001	**0.002**

^a^ Standardized regression coefficients

^b^ continuous variable starting from April 2nd (scored as 1) and ending at April 1st (scored as 365)

## Discussion

This is the first study to demonstrate a linear inverse association between relative age and emotional well-being in early adolescents using a large individual-level dataset. Furthermore, it suggests that academic performance and bullying mediate any effect of younger age relative to peers on well-being.

The result of this study is consistent with previous studies, which showed the relative age effect on mental health in adolescence [5–9]. The previous studies compared the earliest and latest birth month [5, 8] or divided students into three groups and compared them [6, 7, 9]. This study added further evidence regarding the linear inverse relationship between relative age and emotional well-being among early adolescents, though the effect size was very small. A disadvantage in school performance because of relative young age may lead to low self-confidence and self-esteem [24], as well as low emotional well-being. Because disadvantages at school increase as the relative age of children becomes younger, it would be reasonable that the association between relative age and emotional well-being is linear, as observed in this study.

Considering mediation analysis via structural equation modeling, poorer academic performance was suggested as a mediator of relative age effect on emotional well-being. Lower academic performance in relatively younger students observed in this study accords with previous studies [12, 13]. Lower academic performance may lead to lower self-esteem, then to lower emotional well-being in relatively younger students. It is assumed that the participants of this study who were relatively younger than class peers had experienced lower self-esteem for several years since entering primary school.

Being bullied was also suggested as mediating any effect of younger age relative to peers on well-being. Relatively younger students may be both physically and psychologically less mature than peers, which is a risk for being bullied [15, 16]; thus, they may tend to be victims of bullying. The effect of bullying victimization on emotional well-being was stronger in males than in females, possibly because boys are more often subjected to direct physical attacks than girls [25]—that is, they would be influenced by relative age more than indirect attacks.

Several clinical implications can be induced from this study, although the effect of relative age on emotional well-being was very small and other important factors would also affect well-being. First, we should consider flexible choice of academic grade in elementary school. Although the academic grade in elementary school is decided automatically by birthdate without any flexibility in some countries, including Japan, other countries allow parents to choose late entry to school depending on the growth of their children. Second, strategies are required to support relatively young students with low academic achievement in elementary school. Although lectures are given to all students in class at the same time in many elementary schools, different lectures should be considered for groups of students with different academic achievement levels. Additionally, educational strategies should be implemented that do not lower the self-esteem of relatively young students with low academic achievement. Third, professionals working for adolescents should note that relatively young students in their school grades are vulnerable to bullying victimization, which may lead to low emotional well-being. Fourth, family members should also note the association of relative age with bullying and emotional well-being since the involvement of family members who are older than the child is associated with reduced incidences of bullying [26, 27].

This study has several limitations. First, the observed association in this study may not be because of the relative age effect but because of the seasonal effect. Several studies found an association between the season of birth and suicide rate [28–33]. Although several underlying mechanisms were suggested, such as seasonal changes in maternal nutritional status, vitamin D levels, and monoamine metabolism-related genotypes [28], there was no clear evidence supporting this assumption. In fact, many previous studies do not contradict the relative age effect. For example, previous studies in Hungary and the UK showed higher suicide rates in individuals born in the spring and early summer [29, 33, 34]; such individuals could be relatively young at school in those countries where the academic year starts in September. Second, since this study used cross-sectional data, we could not determine the causal relationship between emotional well-being, bullying, and academic performance. It may be possible that lower emotional well-being leads to bullying and lower academic performance. Third, because this study is based on one country, it is not clear whether the present results apply to other countries. Fourth, we did not collect academic data from teachers, but we measured observational academic achievement from parents’ reports. Some parents may not recall their children’s report card nor know other children’s scores. Moreover, parental responses may be influenced by their expectations. Fifth, though self-esteem may play an important role in the association between relative age and emotional well-being, we did not assess self-esteem in this study. Sixth, the response rate in the survey (43.8%) was not very high. The samples might be skewed in terms of higher income levels and a higher prevalence of bullying experiences. Compared with a previous study, prevalence of the bullying experience was relatively higher in this study [35], though this situation might be based on different forms of data collection.

Similar studies examining “relative age effect” will be required in other countries in the future. If a similar finding was shown in the other countries, relative age effect on emotional well-being would be confirmed; if not, differences in educational systems between countries with and without a relative age effect should be examined. Additionally, studies are required for changes in relative age effect along with school grade. Further, studies are needed to examine to which specific types of problems/outcomes relative age is related.

This was the first study demonstrating that relatively younger children in elementary school had lower levels of emotional well-being. Further, this study revealed that academic performance and bullying underpinned the relative age effect. Although the relative age effect was very small, the results of this study suggest the need for strategies to compensate for disadvantages in academic performance and peer relationships among relatively young students in elementary school. Similar research will be required in other countries to confirm the findings or educational systems that compensate for the relative age effect.
